# Pathological and Practical Researches on Diseases of the Stomach, &c. &c.

**Published:** 1829-04-01

**Authors:** 


PATHOLOGICAL AND PRACTICAL RESEARCHES ON DISEASES OF THE STOMACH, &c. &c. By *John Abercrombie*, M.D., &c. pp. 396. Edinburgh, 1828.

But a few months have elapsed since Dr. Abercrombie's Researches on the Pathology of the Brain occupied the attention of our readers, and we are happy in finding that the reception which he then met with has encouraged him to favour us with another visit. His philosophic cast of mind, and his ample opportunities for observation; his jealousy of principles too implicitly received, and his distrust of authority too imperious to be useful; his patience in investigating every circumstance, and his prudence in explaining every fact; his dread of theory, and his fondness for induction, eminently qualify him for medical research, and especially entitle him to unsuspecting credit.

In the present state of our profession such authors cannot be too highly estimated, for it cannot and it ought not to be concealed, that many of the works of this our enlightened age and far-famed country are more indebted for their existence to the urgencies of poverty, or to the luxuriancies of fancy, than to the pregnancy of genius, or the maturation of science; and that for every volume which may be consulted with *safety*, leaving *profit* wholly out of sight, we have five in which *harmlessness* is not their lowest excellency. The expenses of the press have become so trifling, and the mania of authorship so intense; the number of readers has so increased, and the competition among writers has become so active, that students turn authors before their grammar has been half repeated, and authors turn students after their works have introduced them to the practice of their profession. Pompous title-pages have now become modest sign-boards, and octavo volumes merely voluminous announcements that a license has been taken out.

In the present work the same *modus philosophandi* has been pursued as in the former volume;—interesting cases of disease are judiciously collated and carefully detailed;—their leading features during life, and their morbid products after death are attentively noted;—their points of resemblance and distinguishing peculiarities are cautiously traced out, and from these, as data, such inferences have been drawn as seemed to the author legitimate and natural. Thus, while we are presented with his general principles, the materials out of which these principles were constructed are laid before the public, so that if his premises be incorrect, or his conclusions illogical, we have the means in our own power of rectifying both. It is undeniable, that all the certainty of which medicine can yet boast, has been attained by following this method, and the indisputable fact, that all her errors have arisen from sanguine anticipation and untimely inference, will bear us out in the averment, that unless the inductive philosophy be rigidly pursued, and experience alone made the test of what we see and think, she will never be entitled to the name of science, but be ever an undigested and deceitful mixture of facts and fancies. The field of fiction is the Poet's realm, where imagination may wanton with truth and probability; but in medicine, dry and abstract fact must be our only study, and he, who has not the patience of a philosopher to decipher, by slow investigation, the mysteries of disease, need scarcely hope for the endowment of a prophet to reveal them by foresight.

As it will be impossible to do justice to this work in a single article, we intend to confine ourselves, at present, to the Doctor's pathology of the stomach, liver, spleen, and pancreas, leaving his 2d and 3d parts, which are devoted to intestinal affections, for a future number; and, although we thus make a trifling encroachment upon his arrangement; no confusion can arise from the alteration, and the subject of our second article will remain unique.

After making a few preliminary observations upon the anatomy of the stomach, the nature of its coats, and the diseases resulting from derangement in their functions and structure, he remarks;—

"I shall consider affections of the stomach under three classes:

"I. Affections of an inflammatory kind, including ulceration and its consequences.

"II. Affections which more properly come under the class of organic.

"III. Functional affections,—embracing a slight outline of the subject of dyspepsia.

"In an appendix, I shall briefly allude to the affections of the œsophagus—and the duodenum—and to derangement of the functions of the stomach by tumours attached to it externally." 12.

### Inflammatory Affections and Ulceration.

*Acute Gastritis* is generally described among the phlegmasiæ by our systematic writers, but it is a disease of very rare occurrence. In extensive peritonitis, the peritoneal coat of the stomach is sometimes implicated, but the Doctor has never seen an instance of idiopathic gastritis; and athough Dr. Armstrong, in his first fasciculus on morbid anatomy, gives a plate representing inflammatory deposition upon the exterior surface of this viscus, the history of the case is not affixed, and we are not informed whether it was merely an extension of a more general disease, or entirely confined to the stomach.

The disease which we designate gastritis, therefore, is principally, if not wholly seated in the mucous coat, and the symptoms by which it is attended are remarkable for their uncertainty. A man, mentioned by Haller, was seized with pain in the stomach and fever, in consequence of drinking cold water while heated, and died delirious in fifteen days; in another case the chief symptoms were pain and oppression of the chest, hiccup, and difficult deglutition. In both, the stomach was found highly inflamed, and in the former, gangrenous and ulcerated patches were discovered upon its fundus. In a case by Morgagni, the patient complained of anxiety, a sense of fulness in the stomach, and frequent vomiting, which were succeeded by hiccup, delirium, and convulsions; in another, by Storck, a sense of burning in the abdomen, hiccup and intense thirst are specified; and Lieutaud particularizes violent pain in the stomach, distention of the epigastre, vomiting and dyspnœa. But De Haen, Stohl, and Frank, give cases in which the majority of these symptoms were absent, and others exhibiting them all and yet presenting upon dissection no inflammatory appearance. This uncertainty among the older writers has not been much diminished by more recent investigation, and in the present instance the author is disposed to regard as points not yet ascertained,—

"What are the characters exhibited by the mucous membrane of the stomach in the earlier periods of acute gastritis, and in what they differ from appearances which may exist without any symptoms of gastric disease, or take place after death. If we might proceed in any degree upon the analogy of the corresponding affection in the mucous membrane of the bowels, I should be inclined to suppose, that the disease exists under two forms;—that in the one, it is seated chiefly in the follicles or simple glands, in the other, in the mucous membrane itself;—that, in the former case, it would consist, in its early stage, of detached and minute pustules or vesicles, and would terminate at an early period in minute and detached ulcers;—and that, in the other, it would exhibit in its first stage, the appearance of defined portions of the mucous membrane of a red or livid-brown colour, and sensibly elevated above the level of the surrounding parts—these portions afterwards terminating by softening or ulceration, or passing into a chronic state of disease with ulceration, thickening, or fungoid elevations upon the diseased parts. This is in some measure conjectural, but I think we may safely assert, that, in this investigation, nothing can be founded upon a mere general or extensive redness of the membrane, discoloration or increased vascularity,—whether more or less extensive,—venous turgescence, extravasation of blood into the cellular texture, or upon any appearance which consists of mere change of colour, without any decided change in the structure of the part." 16.

But while our facts are few and our knowledge uncertain on the subject of *acute* gastritis, we are not deficient in cases of a *chronic* form, which, from the slow and insidious nature of its progress, is not less destructive to the health and happiness of its victim. In the early stages the symptoms are usually dyspeptic;—acidity, eructation, flatulence, oppression of the stomach after eating, and slight epigastric pain. The patient feels easy when his stomach is empty, and although the appetite is occasionally unhurt, food is often abstained from through fear of its consequences. Vomiting is apt to occur, but only at intervals in the beginning; at a more advanced period it becomes frequent, and the gastric uneasiness increasing, the patient either sinks from exhaustion, or is rapidly cut off by a sudden aggravation of all his symptoms. In some instances there is little or no vomiting, while in others the prominent feature is a copious discharge from the stomach of a fluid like white of eggs; in some there is a constant and most painful feeling of pyrosis, but in many, little will be complained of until the attack occur which proves suddenly fatal. In a case by Andral four pints of a glairy fluid were thrown up every 24 hours, while the woman neither vomited her food nor drink; and Chardel, Pinel, and the author, relate instances in which there was tolerable appetite, scarcely any vomiting, and uneasiness only after food. The Doctor's 4th and 10th cases are good illustrations of the insidious nature of this affection, and prove to what an extent the work of disorganization may proceed without exhibiting any formidable symptoms. The intermitting character of the pain, the absence of vomiting, and the general appearance of health until a short period before death, in the fourth case, indicated nothing more than obstinate dyspepsia; and in the tenth case the patient was never known to complain of his stomach, or to betray the smallest deviation from robust health until 18 hours before death!

A gentleman, æt. 53, complained of epigastric pain, which always came on after dinner, and lasted an hour or two. He was cupped over the stomach, confined to a regulated diet, and took the oxyde of bismuth, by which means he was restored to health, and continued so for six months, when the above symptoms returned, and, after a variety of unsuccessful measures, obliged him to come to Edinburgh in June to seek relief. His general aspect was, however, even then, that of health, his pulse natural, bowels easily regulated, had no vomiting, and, although the pain which he experienced after taking food was severe, at other periods he was easy, and pressure over the epigastrium discovered no particular tenderness. After ordinary treatment for a few days, he absented himself from town for a fortnight, and upon his return was found labouring under dropsy. He now declined rapidly in strength and flesh, said that he felt as though there was no room in his stomach for food, and refused almost every form of nourishment. His pulse, however, continued calm, he only vomited slightly a few times, and, except a small tumour discoverable to the right of the umbilicus, there was no appearance of organic disease. Notwithstanding a variety of treatment he gradually became worse, and about the 25th of July died.

*Inspection.* Much water in the abdomen;—peritoneum thickened, firm, and covered with miliary tubercles;—stomach much contracted, coats thickened and hard, on its exterior a mass of tubercular disease, and on its internal surface an ulcer of the size of half a crown. The omentum was hard and tuberculated, a portion of which adhered to the right parietes of the abdomen and formed the tumour hinted at above, and another part was so intimately attached to the stomach that they seemed blended into one mass. The duodenum was healthy, the liver pale and small, but upon the whole little affected, and there were a few adhesions among the small intestines.

A servant girl, æt. 21, while engaged at her work in the morning was seized with excruciating pain in her belly, sickness and vomiting; she was bled three times in the course of the day,—once, to syncope,—purgatives were given but did not operate, and the bowels were moved by an enema. The abdomen, nevertheless, became tense and tender, the pulse feeble and rapid, every thing she took was vomited, and she expired about two, next morning.

*Inspection.* Extensive peritonitis, — peritoneal cavity distended with air, and contained some fluid like that she had swallowed, bowels coated with a puriform matter,—in the middle of the small curvature of the stomach there was an opening of about one third of an inch in diameter, around which the coats of the stomach were half an inch thick, and internally, corresponding with this opening, there was an ulcer half an inch in diameter, which had extended through the thickened coats to the peritoneum, that had given way in the fatal attack.

The symptoms and effects above described evidently proceeded from chronic inflammation of the mucous membrane, which frequently commences in a small portion, is very tedious in its progress, and seems to subside and return after various intervals, until it occasions more permanent disease. Ulceration, which is usually the cause of the most urgent symptoms, may exist in various forms, of which the following are the most important;—

"A small defined ulcer of limited extent, with evident loss of substance, and rounded and elevated edges, varying in extent from the size of a split pea to that of a shilling. We may find only one such ulcer, every other part of the stomach being in the most healthy state; or we may find that there has been a succession of them, some of them cicatrizing, and others appearing, while the health of the patient gradually sunk under the disease, which after all may be found to have been of no great extent. In the cases of this first class, there is no general disease of the coats of the stomach, the ulcer being confined entirely to the mucous membrane, or perhaps to the follicles.

"Ulcers like the former, of small extent, perhaps the size of a shilling, but complicated with thickening and induration of the parietes of the stomach, perhaps to the extent of a crown-piece or more around the ulcer, all the rest of the stomach being perfectly healthy.

"Extensive irregular ulceration of the inner surface of the stomach, generally complicated with thickening and induration of the coats, and fungoid elevations.

"In some cases there is no actual ulceration,—the prominent morbid appearance being a thickened state of the mucous membrane to a greater or a less extent. The thickened portion in this case may be of a pale ash colour, or of a brown colour, or of a dark colour with the characters of melanosis; and these appearances may be farther complicated with thickening and induration of all the coats of the stomach at the part affected, and perhaps adhesion to some of the neighbouring organs. In other cases again portions of the mucous membrane have been found softened or entirely destroyed." 20.

An example of the third variety will be seen in the case we have already extracted, and, as specimens of the first two, we adduce the following:—

A gentleman, æt. 50, who had been long in the West Indies, began to complain, about two years before his death, of dyspeptic symptoms, which did not, however, assume a serious aspect till the Winter of 1824, when intermitting pains in the region of the stomach, which were sometimes dull and occasionally acute, vomiting, impaired appetite, and loss of strength alarmed him. Towards Spring he improved; during Summer he complained most of anorexia, a dull pain across the epigastre, and debility. His aspect became languid, pulse weak, powers exhausted and, although no organic disease could be detected by the most minute examination, he died about the middle of June in an incoherent and lethargic state.

*Inspection.* Stomach large, but externally healthy;—on the inside two or three small round ulcers with inflamed margins were observed about the middle of the small curvature,—higher up nearer the cardia there were many white or ash-coloured spots, like the marks of variola, which bore all the characters of cicatrices. The coats were neither thickened nor indurated, and no further disease was visible either in the stomach or remaining viscera, excepting an old adhesion of the apex of the heart to the pericardium.

A woman, æt. 45, was occasionally addicted to pain and oppression at the stomach, her appetite was variable, and she vomited at irregular intervals. For some time she had been emaciating, but her complaint only assumed a decided character about two months before her death, when the pain across her stomach became severe, vomiting frequent, pulse small and quick, and bowels very loose. She was first seen by Dr. Begbie about a fortnight after the appearance of these symptoms, and she died exhausted six weeks afterwards.

*Inspection.* The coats in the small curvature of the stomach were thickened to three fourths of an inch, and so indurated as to form a hard mass of three inches length, which, when cut through, presented a white cartilaginous structure, except on its internal surface, which was in a state of soft white fungous ulceration. The rest of the stomach was healthy, and the mucous membrane of the intestines contained many vascular patches, but without any structural alteration.

Chronic gastritis may destroy life by producing gradual exhaustion, by giving rise to hæmorrhage, and by ending in peritoneal inflammation. The three patients, whose histories we have adduced, evidently died in the first way; and the following case proved fatal by hæmorrhage from the ulcer.

A gentleman, æt. 40, who had been for some time troubled with dyspeptic symptoms, was suddenly seized in his counting-house with a feeling of extreme weakness, and soon after vomited a large quantity of fluid resembling ink. On the next day he continued very sick, and discharged occasionally by vomiting and stool the same kind of liquid. On the third day he was attacked with pain in the stomach, followed by vomiting of pure blood to several pounds, when he was seen by the Dr. for the first time. He was then extremely pale and exhausted, and complained of faintness, and, although every attempt was made to rally him, he died in the night.

*Inspection.* Stomach was very large, but exhibited no signs of disease, except in the small arch, where there was an ulcer upon the internal surface, of half an inch in diameter and more than a quarter of an inch in depth, the bottom of which was occupied by a fungous mass of a dark brown colour. No other disease could be detected in any organ.

When this disease proves fatal by perforating the stomach, we find on inspection two important modifications of the morbid appearances; in the one there is ulceration without further disease, in the other the coats are much thickened, the ulcer seems to have penetrated the condensed substance, and to have cicatrized at the edges, leaving a defined cavity with smooth walls having the peritoneum for its floor. It is obvious that this form of disease must be of long standing, and that the fatal event ultimately depends on rupture of the slender peritoneal covering. But even in cases so extreme, life is occasionally protracted for a period, by the formation of adhesions between the edges of the ulcer and some of the neighbouring parts, most commonly the liver, in such a manner, that the adherent portion of the liver supplies the want of continuity in the stomach, and thus an artificial floor is given to the former perforation. An interesting specimen of such contrivance will be recognized in the succeeding history :—

A gentleman, æt. 60, had suffered for a considerable time from an intense feeling of pyrosis, occasional attacks of vomiting and epigastric pain, on account of which he was often obliged to leave the table during meals. He, however, improved so much upon a regulated diet that his symptoms disappeared for a considerable time, and his stomach regained its healthy functions. About a fortnight before his death, after experiencing one of those slighter attacks to which he had been addicted, he became confined to his house, but was able to attend to business until the day before he died. Upon that day, he was suddenly seized with excruciating pain at the pit of the stomach, attended by vomiting, coldness of the body, and a small frequent pulse. Through the night his symptoms continued unabated, and next day his abdomen became distended and tender, his powers rapidly gave way, and he expired 30 hours after the attack.

*Inspection.* On the posterior surface of the stomach, near the pylorus, there was a portion of its entire substance larger than a shilling completely destroyed, but the hole was patched up by adhesions between its margins and the surface of the liver. Below this spot, and nearer the pylorus, there was an ulcer of about half the size, through which a perforation had taken place to such an extent as would have transmitted a full grown quill. The contents of the stomach, escaping by this passage, had produced extensive peritonitis, under the immediate influence of which the patient sunk.

Nature had in this instance been evidently struggling with the inroads of disease, and had succeeded in procuring a respite for her victim by patching up a former ulceration, and, had not the second ulcer been unfavourably situated by its low position for the formation of a fresh adhesion, death might have been again warded off, as the rest of the stomach was perfectly healthy. M. Gerard, in a Memoir *Des Perforations spontanées de l'Estomac*, has detailed 17 cases of perforated stomach, where the mode of attack and morbid appearances corresponded with the specimen now given ; others are recorded in the *Medico-chirurgical Transactions*, by Dr. Crampton and Mr. Travers, and the author cites instances from the *Journ. Gen. de Med.* for 1821, the first volume of the *Arch. Gen. de Med.* and the celebrated case of Admiral Wassenaer, as narrated by Boerhaave ; but our limits restrain us from being more particular.

The insidious nature of this disease renders its diagnosis obscure and difficult, for since its symptoms, even to the last, differ but little from those of dyspepsia, the generality of practitioners see no reason for suspecting the internal mischief which is silently going on. Some doubt, however, may be entertained when pain is complained of after taking food, especially if it be referred to a particular spot which is tender under pressure. When this pain continues until vomiting occur, when there is an intense feeling of pyrosis, and when the patient weakens and emaciates, or complains of tenderness of his tongue and throat, the language of the preceding symptoms will be more intelligible, and some disease of the mucous membrane of this organ may be dreaded. This disease may either be chronic inflammation of varied extent, or actual ulceration ; the external phenomena attending each are little different, and the labour of drawing a diagnosis, were it possible, would scarcely be recompensed by the additional light which it might throw upon our plan of treatment. When it is detected at an early period, topical bleeding, blistering, issues, and tartar emetic ointment are our most hopeful remedies. Little can be expected at this stage from internal medicines, beyond slight aperients. When it is more advanced, the oxyde of bismuth, lime-water, nitric acid, small opiates combined with mucilaginous substances, astringents, the sulphate of iron, and nitrate of silver, may be beneficially given under appropriate circumstances. Food must be small in quantity and mild in quality, consisting principally of farinaceous matter and milk; all stimulating liquors must be prohibited, and the patient should abstain from much bodily exercise.

"Whether the disease can be cured, after it has advanced to ulceration, must indeed remain in some degree a matter of doubt; because in a case which has terminated favourably, we have no means of ascertaining with certainty that ulceration had existed. In some of the cases, however, which have been described, we have seen every reason to believe that some of the ulcers had cicatrized, though the disease had afterwards gone on to a fatal termination; and from what we observe in the intestinal canal, we can have little doubt that simple ulceration of the mucous membrane may cicatrize. I am satisfied that I have seen the cicatrices of such ulcers when the patient has died of another disease, after having been for a considerable time free from any symptom in the bowels." 50.

In addition to acute and chronic gastritis the author includes among inflammatory diseases of the stomach, *Inflammation of its mucous coat, combined with a general inflammatory state of the whole mucous lining from the pharynx downwards*, *Ramollissement* and *Diphtherite*. The first affectionis peculiarized by a sense of rawness in the mouth and throat, redness of the pharynx interspersed with aphthous crusts, tenderness of the epigastre, and uneasy deglutition. In some cases vomiting occurs, in others diarrhœa, and in some both vomiting and diarrhœa. Lime water, or equal parts of it and a strong decoction of quassia, have been found useful; small opiates will be necessary, and a little wine may be added to a mild diet when the strength has been much impaired.

Somewhat similar to this disease is *Diphtherite*, a disease described at considerable length by Bretonneau, and regarded by the French as a modification of croup. But there is every reason to believe that it is primarily an affection of the mucous membrane of the fauces and œsophagus, which may extend in one case to the stomach, in another to the larynx. It chiefly affects children, and, although not frequently met with in this country, it prevailed as an epidemic in Edinburgh in the Summer of 1826, when an account of it was given by Dr. Hamilton jun. The symptoms which it usually assumes are a deep redness of the tonsils or velum with aphthous crusts, but without either swelling or ulceration. When these crusts are removed, the subjacent membrane appears red but entire, and in a few hours the aphthæ are re-produced. The lining of the lips, mouth, and nose is generally excoriated, the gums spongy and bleed, and in some cases the entire mouth becomes inflamed as from the operation of mercury. There is little fever, but great prostration of strength; the whole habit is irritable and disposed to disease, and the affection occasionally extends along the œsophagus to the stomach, producing epigastric tenderness, vomiting, and sometimes diarrhœa. When it proves fatal the larynx is generally implicated; and when this termination does not occur, the disease seems to run a certain course, over which medicine has as yet suggested no remedy. It is distinguished from cynanche maligna and sore throat in scarlatina by the absence of ulceration, and the sketch which we have drawn of its beginning, symptoms, and progress, will prove its difference from croup, from which it should be carefully distinguished, as the latter, when in an idiopathic form, admits of and requires treatment which would be destructive in the former. In the epidemic of 1826 it was necessary to support the strength, and some benefit was obtained from vegetable and mineral acids, free ventilation, a careful regulation of the bowels, and frequent sponging of the body with tepid vinegar and water. Hamilton advises the acetate of lead internally and in gargle; Bretonneau confides in the free use of calomel, and touches the aphthæ with equal parts of honey and hydrochloric acid; and our author thinks that calomel, combined occasionally with opiates, and, when there is much affection of the stomach, bismuth and lime water, are our most effectual remedies. When the larynx is affected the danger is extreme, for bleeding cannot in general be borne, and blisters are apt to produce gangrene.

Many instances have occurred, in which the stomach has been found after death perforated by large irregular openings, without any symptoms having appeared during life to indicate gastric disease. Hunter ascribed their formation to the action of the gastric juice, but we have reason for believing that they may arise from other causes, as Dr. Gairdner has brought forward many cases which exhibited during life obscure symptoms referrible to the stomach.

"Upon the whole, the conclusion, in regard to this singular affection, seems to be, that it takes place after death; that it has been in some cases preceded by disease of the stomach; but that, in others, there has been no ground for believing the existence of any such disease. It is certainly not an appearance on which any pathological principle can be founded, in regard to previous disease; and this is a point of the utmost consequence, especially in reference to the judicial examination of bodies in cases of suspected poisoning. For various interesting details in regard to it, I refer to Dr. Gairdner's Essay.

"Nearly the same observations seem to apply to the ramollissement of the mucous membrane of the stomach, on which much attention has been bestowed by some of the French writers, particularly in a very interesting memoir by M. Louis.* This appearance consists in portions of the mucous membrane being found in a soft state like semi-transparent mucus, in general without any other disease of the parts. In nearly all his cases, it occurred in persons who had also been affected with other diseases, chiefly phthisis; and they had complained for some time before death of pain and heat in the epigastric region, with loss of appetite, nausea, and occasional vomiting. It is, however, to be observed, that, in a large proportion of the cases described by M. Louis, there existed some other disease capable of accounting for derangement of the functions of the stomach and uneasiness in the epigastric region, such as disease of the liver and spleen, and ulceration of the mucous membrane of the bowels; and farther, that M. Louis himself shows this ramollissement of the mucous membrane existing where there had been no symptom referred to the stomach. Upon the whole, there seems reason to doubt whether this is to be considered as an appearance on which can be founded any principle in pathology."

### ORGANIC DISEASES OF THE STOMACH.

In most of the cases above given there was organic disease, but as the mucous membrane seemed to have been the first seat of action, the author refines a little for the sake of arrangement, and devotes the present section to some affections more purely organic; as "Induration and thickening of the Coats of the Stomach," "Diseases of the Pylorus," and "Diseases of the Cardia." In the first of these disorders, which presents very little variety in

" * Louis' Memoires et Recherches Anatomico-pathologiques.

its symptoms, and admits of little treatment, the altered structure assumes in some cases, the character of scirrhus or cartilage, and in others that of tubercular disease. The succeeding case will illustrate both varieties:—

A woman, æt. 56, who had been liable for about a year to distention, acidity, and occasional attacks of acute pain at the stomach, began to be affected a few months before her death with vomiting, which generally occurred after dinner and returned daily. This vomiting became so severe at last that she threw up much blood, and died in a state of extreme emaciation.

*Inspection.* Stomach adhered to all the neighbouring viscera; the cardia and pylorus, with small portions of the adjoining coats were healthy; the large curvature and anterior part were extensively ulcerated, dark, thickened, and perforated at one part to the size of a shilling; the small curvature was quite scirrhous, about an inch in thickness, and, when cut into, white and firm, and about the centre of this scirrhosity, it contained two tumours, of a dark purple colour externally, and internally white, one the size of a pigeon's egg, the other of a hazle-nut; the pancreas was hard; liver tubercular; other viscera healthy.

When disease of the pylorus is in its first stage it may scarcely be recognizable from simple dyspepsia, but when far advanced it is distinguished by periodical vomiting after meals, with a fixed uneasiness in the epigastrium, and examination can commonly detect more or less induration in the region of the pylorus. Cases do, nevertheless, occur, whose symptoms present remarkable deviations from the ordinary combination, and the following history will prove that extensive pyloric disease may exist with a tolerable state of health, and without vomiting or any induration perceptible externally.

A gentleman, æt. 60, came under the Doctor's care a few weeks only before death, and was found in an extreme degree of emaciation, with an exhausted and withered aspect. He was at first affected with frequent but irregular fits of vomiting, which were in a great measure prevented by a regulated diet; but his debility and emaciation increased, and, although the most careful examination could detect no organic disease, and his gastric irritation had almost entirely subsided for several weeks, he died in a state of extreme exhaustion.

*Inspection.* The pylorus was surrounded by a mass of scirrhus, the size of an apple, the internal part of which projected into the stomach; the principal projection of the tumour was backwards, and, by its adhesions, the pylorus was firmly bound down to the pancreas. The rest of the stomach and the other viscera were healthy.

Cases of a similar nature are given from Chardel, the *Journ. de Med.* for 1815, and the 6th volume of the *Med. Observ. and Enq.*, by which it is obvious that these variations from the ordinary phenomena do not depend upon the degree of contraction with which the pylorus is affected, nor, indeed, upon any morbid difference discoverable by dissection. In one instance the pyloric orifice was nearly closed, yet there was no vomiting until three days before death; in another no contraction existed, while there had been frequent attacks of vomiting and pain.

The 20th case we extract as an interesting specimen of *Disease of the Cardia.* A man, æt. 38, after suffering for many years from difficulty of swallowing, consulted the Dr. in the Summer of 1815. He had pain on pressure behind the ensiform cartilage, the articles which he swallowed seemed to stop at a point corresponding with the cardiac extremity of the stomach, and a slight hardness could be felt there. When he was first visited he could swallow fluids, but during Summer the disease increased until he could scarcely swallow any thing, and he died in November from gradual exhaustion.

*Inspection.* A mass of scirrhus, about three inches in length, extended from the cardia along the œsophagus, which nearly obliterated the passage and projected into the stomach, that was otherwise healthy.

### Functional Diseases of the Stomach.

*Dyspepsia.* In health the digestive process does not occupy a longer period than from three to five hours, no gas is extricated in the stomach, and a certain quantity only in the alimentary canal. Magendie's experiments prove that, during its progress, the food is slowly moved backwards and forwards between the splenic and pyloric ends of the stomach, this motion being in an inverse ratio, as to activity, to the quantity of food. When, however, the tone of the stomach has been injured, digestion is more slowly and less perfectly performed. The food remains longer in the stomach than it ought to do, acid gas and ill-digested chyme are the results, irregular muscular action arises from the morbid stimuli thus produced, giving rise to eructation, nausea, and vomiting; or, the stomach yielding to the accumulating products, distention, oppression, and anxiety, are occasioned. In the preceding sections these morbid phenomena were occasioned by structural disease of this organ, but in the present they depend upon simple derangement of function.

"We have much reason to believe, that the muscular action of the stomach may be deficient, so that the alimentary matters remain in it too long, are imperfectly changed, and pass into chemical decompositions. We know the state of the urinary bladder, in which its muscular action is lost or very much impaired, and in consequence of which, it is gradually distended, so as to hold an enormous quantity of fluid; and when emptied by the catheter, it does not contract equally, as in the healthy state, but falls flat like an empty bag. A state analogous to this we not unfrequently see in the stomach on dissection,—a state in which it appears much enlarged, and collapsed by flattening, without healthy contraction.

"2. There may be a deficiency of the corresponding and harmonious intestinal action, interfering with the second stage of digestion, and giving rise to imperfect chylification and various morbid actions in the upper intestines.

"3. The various fluids may be deficient in quantity, or morbid in quality, so as to derange the process in various ways. We have grounds for assuming that the fluids of the stomach may be in a morbid condition, without actual disease of its coats. We see in certain cases a fluid brought up by eructation in large quantities, in a morbidly tenacious state, quite different from the healthy appearance of the fluids of the stomach; and we have reason to believe, that similar changes may take place in the other fluids concerned in digestion, particularly the bile.

"4. If the mucous membrane be morbidly irritable, the muscular coat will probably be too easily excited to action, and a different state of things will arise. If this occur in the stomach, the articles will not be allowed to remain in it a sufficient time for healthy digestion; but after producing much uneasiness, they will either be rejected by vomiting, or propelled in a half-digested state into the intestines, there to prove a source of new irritation. This is probably the state to be afterwards more particularly referred to, in which animal food produces much uneasiness in the stomach, often followed by vomiting; but in which digestion goes on in a healthy manner, on a regimen restricted to farinaceous articles and milk. If the irritability occur in the intestine, the articles may undergo their proper change in the stomach, but will be propelled too rapidly through the intestinal canal, without time being afforded for the complete process of healthy chylification; and, accordingly, in many affections of the stomach and bowels, we see articles, even of the most digestible kind, pass through partially digested, or sometimes entirely unchanged." 71.

Several dietetic rules arise as inferences from the above pathological positions. In the first place, since the action of the stomach is in an inverse ratio to the quantity of its contents, dyspeptics should be restricted to such a quantity of food as the stomach shall be found capable of effectually digesting. Secondly, substances of the easiest solution should be chosen, and the more nearly they resemble each other in this property, the more suitable will they be for the weakened stomach, and for the formation of healthy chyme. The articles of easiest solution seem to be solid animal food and white-fish, both plainly dressed. Vegetables are less digestible, and fatty, tendinous, or cartilaginous parts are least adapted for a dyspeptic stomach.

If in health digestion occupy about five hours, and if this period be prolonged by dyspepsia, a longer interval should be allowed between each meal, and the depressed energies of a debilitated function should not be annoyed during their operation by any unnecessary infringement. With our author we believe, that more attention should be paid to the quantity than even to the quality of food given to dyspeptics, and when we are daily pampering our appetites by mixtures of the most heterogeneous nature devoured in overwhelming volumes, we have more reason to be astonished at the resources of Nature than at the prevalence of disease. No plan could be devised for impairing the appetite and injuring digestion more certainly and more speedily effectual than mixing up in the same repast a great variety of dietetic articles, of different degrees of solubility. The variety of the dishes invites desire, desire gives a transient excitement to the appetite, the appetite solicits the action of the stomach, the stomach is encouraged to receive what it has not power to digest, indigestion produces fermentation, fermentation acidity and all the miserable sequelæ of a gluttonous debauch. Articles, which cannot be endured as a part of mixed diet, may be tolerably digested if taken singly, and the stomach, which may fail in concocting a complicated compound, shall digest with ease the individual simples of which it is composed.

"In the regulation of the diet for all affections of the stomach, however, strict attention must always be paid to the nature and source of the disease. Animal food is in general the most digestible, but there are many cases which depend upon an irritable state of the mucous membrane, in which the diet found to be beneficial or even necessary, is one restricted to farinaceous articles and milk. The higher degrees of this affection, in which the disease amounts to inflammation of the mucous membrane, have already been referred to; but there appear to be modifications of it, which, without assuming this formidable character, have a similar effect on the functions of the stomach, and require similar treatment, especially in regard to diet. The subject is one of great interest, and opens a most important field of observation to him who, renouncing a mere empirical treatment of dyspeptic affections, shall direct his attention to the important varieties in the nature and source of the disease. Such a person will be astonished to find the improvement which is made in certain cases, under a diet restricted entirely to rice, arrow-root, or bread and milk, with total abstinence from all stimulating liquors, after the patient had spent years of wretchedness upon animal diet, with wine or brandy and water, and the usual round of stomachic remedies. Other cases again agree better with animal diet in very small quantity, and the moderate use of stimulating liquors. The diagnosis is often difficult, and must be guided more by the judgment and attention of the practitioner, than by any general rule. This subject has been well illustrated by Dr. James Johnson, in his treatise on Morbid Irritability of the Stomach." 74.

The Dr. advances nothing new in his medical treatment of dyspeptic complaints, but mild and regulated intestinal action, by various combinations of gentle aperients with tonic medicines, is strongly recommended. Mercury is reprobated, except in cases complicated with hepatic disorder, and, when the desired effect can be attained by any other means, it ought not to be employed in any form or quantity.

*Gastrodynia.* Pain in the stomach may occur in four different forms; 1st, when the stomach is empty, probably from acrimony of its juices, when absorbents and alkalies will be useful; 2dly, when the stomach is full, probably from inflammation, or irritability of its mucous membrane, to the treatment of which we have before alluded; 3dly, when the pain commences about two hours after food, and continues for some time, probably from duodenal inflammation, or morbid sensibility. This variety has been generally confounded with hepatic disorder, from pain and tenderness being betrayed under pressure by the right hypochondrium; but the intermitting character of the affection, and its appearance at a certain period after meals, will sufficiently distinguish it from diseased liver. The Doctor strongly recommends two grains of the sulphate of iron, one of aloes, and five of aromatic powder, taken thrice daily; or oxyde of bismuth combined with rhubarb and lime-water, and small opiates are frequently useful. If, however, the attack be severe, topical bleeding and blistering must precede these remedies, and be accompanied by a spare farinaceous diet. Lastly, the pain may occur at various intervals after food, and continue for uncertain periods in violent paroxysms, which are usually attended by a feeling of anxiety, distention, and restlessness, and in females are frequently complicated with hysterical symptoms. It seems to depend upon flatulency, and is most effectually treated by injections and carminatives.

"There seem to be some other modifications of pain in the region of the stomach, not referable to any of these classes. Among these may be reckoned a pain which affects persons of a gouty habit, and may occur either in the form of severe and sudden paroxysms, or as a more continued pain going on for many days together. It seems in general to be most relieved by stimulants, combined with alkalies and small opiates; but it requires to be carefully attended to, and treated by topical bleeding and blistering, if it do not soon give way. There is also a violent affection of the stomach, occurring chiefly in females of an irritable habit, and assuming a spasmodic or neuralgic character. It seems in general to be relieved by opiates combined with absorbents or alkalies. All these affections of the stomach, however, should be watched with attention, for several remarkable examples have been given which show that they are often connected with chronic inflammation or ulceration, and that they may be very rapidly fatal, without having assumed any formidable character till the fatal attack." 78.

It has been of late quite fashionable to ascribe all these gastric derangements to mucous inflammation, (*gastro-enterite chronique*,) and to treat them as arising from the same cause, and removable by the same remedies. But we refer those of our readers, who may be unsettled upon this subject, to the work of Barras *sur les Gastralgies et les Enteralgies* for a most masterly exposé of one of the most absurd hobbies that ever issued from the schools of France.

In obstinate *pyrosis*, lime-water, bismuth, stimulants, as garlic and benzoin, acids, especially the nitric, and blistering, may be occasionally beneficial; but no rule can be given for discriminating the cases adapted to each. If the fluid thrown up be glairy and copious, it may proceed from a thickened state of the mucous membrane; but if it be tinged and acrid, ulceration may be suspected.

*Hæmatemesis* may appear as a symptom of ulceration, or a varicose state of the vessels of the mucous membrane of the stomach. In many instances, however, it occurs and may even prove fatal where there is no organic disease. Some constitutions are liable to periodic attacks of it, and females, whose menstrual function is deranged, are especially addicted to vomiting of blood. If the strength have been much exhausted by the attacks some restorative in small quantity must be given, and a return of the hæmorrhage guarded against by such medicines as acids, acetate of lead, muriated tincture of iron, bismuth, alum, kino, and refrigerants.

Perhaps the most serious, because the most alarming to the patient, of all the consequences of disordered stomach, are *sympathetic affections of the heart.* They appear in various forms, from the most trifling palpitation to the most irregular action, and not unfrequently assume all the characters of organic disease. Sometimes the patient is sensible of a trembling motion of the heart, such as is caused by surprise, which only endures for a moment, and is accompanied by an intermission of the pulse, but may frequently return during the same day, or be rapidly repeated for the space of an hour. In other cases the palpitation is more regular, and continues without any remission for an hour or more, occurring at, and remaining for, uncertain periods. Sometimes these fits shall continue for several days, without causing any intermission in the pulse of the heart or arteries; and, in others, there is merely an increased frequency in the heart's action, producing a quick but regular pulse, and accompanied by a feeling of anxiety, which continues for periods of uncertain length.

"Betwixt the various forms of this affection and disease of the heart, the principal diagnosis consists in the pulse being regular, and the action of the heart natural, during the intervals between the attacks,—in an obvious connection with disorders of the stomach, and relief by treatment directed to that organ,—and, particularly, by the symptoms being most apt to occur while the patient is at rest, especially after meals,—not being increased by bodily exercise, but rather relieved by it,—and not being excited by such bodily exertion as we should naturally expect immediately to influence a disease of the heart. The affection is always very alarming to a patient, and sometimes perplexing to the practitioner; for, from the permanency of the symptoms, they certainly often assume, in a great degree, the character of disease of the heart, and may even exhibit some of the stethoscopic signs, particularly the *bruit de soufflet.* There is, also, in many cases, a considerable degree of dyspnœa, and sometimes there are paroxysms of it of considerable urgency." 83.

Out of several cases detailed, as illustrative of the above varieties of disordered action in the heart, we select the following as a specimen :—

A gentleman, æt. 48, was attacked daily with paroxysms of palpitation of the heart and intermission of the pulse, which were sometimes repeated thrice in the 24 hours, and continued about an hour at each visit. During their continuance the respiration was laborious, yet walking could be indulged in without aggravating the symptoms; but when they terminated, the heart, pulse and breathing became perfectly regular. His digestion was imperfect, stomach easily deranged, bowels slow, and stools unhealthy. The paroxysms came on, in general, soon after food, but they occasionally occurred at other seasons. A great variety of medicines and forms of diet were employed, London and Cheltenham were visited without benefit, and, although he sometimes emaciated rapidly, and rapidly improved, the affection of his heart remained unchanged in character and unabated in degree. After two years and half were spent in this state of health, all his symptoms at last subsided under the use of vin.

colchici in very moderate doses. It at first acted so strongly as a purgative, that only ten drops could be taken twice a day, but no attempt is made to explain its *modus operandi.*

The influence, which a dyspeptic stomach exerts over the brain, is too notorious to require either proof or illustration. The following history will, however, be perused with interest, as illustrative of the connexion which maintains between gastric, neuralgic, and paralytic disorders; although very anomalous in its symptoms, it seems a compound of the three.

A dyspeptic gentleman, æt. 50, used to be seized, at various times of day, and without any premonition, with an uneasy feeling in the epigastric region, accompanied by a very loud sound, which was the constant signal of an attack of pain in some part of his lower extremities, generally on the inside of the thigh, a little above the knee. This pain, which was so acute that he insensibly grasped the part with both hands to alleviate his sufferings, was followed by a convulsion of the limb, which lasted only for a moment, but returned with the return of pain and epigastric uneasiness. These paroxysms occurred frequently in the day, and in the night he had frequent starting of his limbs. His digestion was bad, bowels confined, and stools unnatural. At one period his extremities became so feeble that paralysis was dreaded, but nothing could be discovered to account for such symptoms. The affection continues to recur in a diminished form, and the only measures which seemed to afford relief were mild purging and moderate opiates.

*Dysphagia.* Since Dr. Monro's work upon the gullet, little has been added to our knowledge of this affection. It may arise from enlargement of the epiglottis and diseases of the larynx, which may, in general, be distinguished by cough and dyspnœa attending the difficult deglutition;—paralysis of the œsophagus, which is usually connected with cerebral and spinal disease, and is much benefited by electricity;—stricture from thickening of the mucous membrane of the œsophagus, or more extensive contraction with ulceration, sometimes arising from inflammation produced by swallowing improper bodies, and sometimes appearing without any perceptible cause; tumours within or upon the exterior surface of the œsophagus, as polypi, enlarged bronchial glands, collections of matter behind the gullet, and aneurism of the aorta;—diseased cardia, which we have previously noticed, and spasmodic stricture, according to Monro, or, as our author prefers considering it, morbid irritability, or inflammatory action of the mucous membrane of the œsophagus. We shall here subjoin a very interesting case of œsophageal inflammation, which, in many points, resembled croup.

A gentleman, æt. 26, (Dr. A. seems to be one of those favoured few, whose patients are either ladies or gentlemen,) complained of his throat, and had a glandular swelling on the right side of his neck. His voice was hoarse and had a peculiar sound, the fauces were of a bright red colour without much tumefaction, but contained several aphthous crusts. Within a few days he became feverish and was confined to bed, but after active treatment for a week his fever abated, and he was left, as to his first symptoms, in the same state in which he was found when the Doctor was consulted. His fever soon returned, however, in a typhoid form, he had some dyspnœa and difficulty of swallowing, which induced cough and vomiting, during which considerable quantities of a soft membranous substance were brought up. His strength rapidly exhausted, and, without any other palpable alteration in his symptoms than a return of natural deglutition for twelve hours before death, he expired at the close of three weeks after the appearance of the disease.

*Inspection.* Pharynx and part of the epiglottis were covered with a soft adventitious membrane, pieces of which were found lying detached within the larynx. A similar membrane was traced along the entire tube of the œsophagus to the cardia, near which it lay loosely attached, "forming a soft continuous mass about one-third of an inch in diameter, and with the œsophagus closely contracted around it." Every thing else was healthy.

*Diseases of the Duodenum.* Our author concludes his observations upon the pathology of the stomach with a few remarks on diseases of the duodenum, upon which little is known with any degree of certainty. We have already alluded to that form of gastrodynia which occurs a few hours after meals, and probably arises from irritation occasioned by the food in its passage from the stomach over the irritable or inflamed surface of this intestine; and it is natural to suppose, that in all serious affections of this organ, one of its leading and most peculiarizing symptoms will be a feeling of uneasiness, more or less intense according to circumstances, commencing at a certain period after meals, and terminating when the stomach has been completely emptied. In a case, related by Dr. Irvine, (Philadelphia Med. Journ. of 1824,) the pathognomonic characters of diseased duodenum are well illustrated. The patient was addicted to attacks of pain and vomiting, which at first returned after long intervals, but soon became more frequent. For three or four hours after food he felt easy and natural, but after that period pain came on, vomiting followed, and the attack continued until the stomach had discharged its burthen. In about six months he died, and, on inspection, the stomach and liver were found healthy, while the duodenum was hardened, enlarged, and studded with tubercles of varied size. In its cavity there were ℥iv. of pus, and upon its internal surface a considerable extent of ragged ulceration. This patient died exhausted; but ulceration of this intestine may prove fatal by perforating its coats and inducing peritonitis, as we have already shown while upon diseases of the stomach. The *Nouvelle Bibl. Medicale*, for 1828, contains a case of this kind. A man, æt. 27, who had been for some time afflicted with epigastric pain, succeeded by diarrhœa, nausea, and loss of appetite, was one day suddenly seized, about three hours after dinner, with excruciating pains in his epigastric region, which soon spread over the abdomen, and caused death in the course of the following day. On dissection, the peritoneum was inflamed and contained much gas and fluid, the stomach was unaffected, but an oval ulcer was discovered in the beginning of the duodenum, of three or four lines in diameter, with rounded edges, and so deep that it seemed to have been floored by the peritoneum merely, which had given way during the last attack, and, by allowing the food to be extravasated, occasioned peritonitis; near this ulcer there was a more superficial one of the same size, which appeared to be confined to the mucous coat.

### Pathology of the Liver.

In the preceding sketch of the Doctor's Pathology of the Stomach, it will be seen that the author's attention has been more devoted to an elucidation of its morbid conditions than to the means by which these conditions may be either prevented or removed; and in the pages which succeed, the same plan has been followed, the same object has been pursued. The peculiarities of a British climate prevent us from collecting much experience upon acute diseases of the liver, and its chronic affections are so insidious and obscure that they are neither easily detected nor relieved. But, were we to be guided by the popular dogmata of the present day, the liver should be the seat of almost every disorder, and whether our brains be muddled or our lungs diseased; whether we writhe under the convulsions of neuralgia, or sleep under the apathy of palsy; whether we eat with voracity, or languish with dyspepsia; disordered bile, or unhealthy liver, is the never-failing solution of the most opposite and perplexing symptoms. This hepatic mania has been probably imported by our oriental visitors, who return to us with rotten livers and wretched stomachs —the staple commodities of the East; but we know of no fact which can establish either the contagious or hereditary nature of such diseases, and the success of a blue-pill and a black draught, of mercurial friction and a few leeches in a dyspeptic Indian habit, goes but a short way in vouching either for their adaptation or effect in every variety of constitution and form of disease. The liver has unquestionably much influence over the functions of the stomach, and its condition can sustain no important change which shall not be announced by some gastric symptoms; but, to use the language of Dr. A. by whom our views are ably supported,—

"I must at the same time confess my suspicion, that it has become a kind of fashion to refer symptoms to morbid conditions of the liver, without any good ground for considering them as being really connected with that organ. This is so common in the modern phraseology of medicine, that it seems a very delicate task to start a doubt in regard to a doctrine so generally received. But as a practical man, anxious to be guided by observation alone, there are three classes of facts which have appeared to me worthy of much attention in reference to this subject; namely, 1. That I frequently see such complaints get well under very mild treatment, as regulation of the bowels, and a little attention to diet; 2. That I have seen such patients put through long and ruinous courses of mercury, without any benefit, and afterwards found the complaint removed by a course of mild laxatives; and 3. That I have known patients die of other diseases, while these alleged affections of the liver were going on, without being able to discover in the liver, upon dissection, the smallest deviation from the healthy structure. I am ready to admit, that in such an organ as the liver there may be morbid actions which do not leave any appearance that can be discovered on dissection, though they may be the source of uneasy sensations and derangements of function. But such actions, if they leave no trace of their existence, must have been of a very temporary kind. If the symptoms have been of any considerable standing, we are certainly entitled to look for some trace of disease, or else to doubt whether the liver was really the seat of the disorder,—particularly if the symptoms were of such a kind as might with equal plausibility be referred to other sources, such as disordered conditions of the stomach or bowels, especially the duodenum or the arch of the colon." 320.

The Dr. arranges hepatic disease into *acute and chronic*, and while he is aware that such a division is liable to serious objections, he would rather violate minute nosology than injure the interests of practical truth; and, as his object is to confine himself almost entirely to the results of his own experience, the liver diseases of this country have been chiefly attended to, with occasional observations on those of India, when any light can be shed by such references on the nature or treatment of the maladies he has himself witnessed.

### Acute Diseases of the Liver.

*Inflammation of the liver* is marked by different symptoms according to the different portions inflamed;—when it is confined to the peritoneal coat the pain and fever are more severe than when the parenchyma is affected; if it exist in the convex surface, pneumonic, if in the concave, gastric symptoms prevail, and icterus is a much more frequent consequence in the latter instance. Simple *peritonitis of the liver* is very rare, inflammation of the hepatic peritoneum being usually combined with that of the entire membrane; and it sometimes happens that parenchymatous inflammation of this viscus produces symptoms as acute as those which are supposed to indicate more superficial disease. Hepatitis may terminate variously;—it may prove fatal in the inflammatory stage, it may pass into suppuration, produce softening of the parenchyma, or end in chronic disease.

"As some of the appearances, however, which will be referred to under these heads, have not been absolutely ascertained to be terminations of inflammation of the liver, I shall not describe them under this arrangement; but, following the course which I have already proposed, I shall simply refer to them in the general investigation of the actual morbid conditions which we find in the liver after death." 323.

What is frequently called *congestion* in this viscus, seems to our author to be nearly allied to inflammation, and the dark or melanotic aspect which it is seen to assume in India he deems to be its effect. This view is strongly countenanced by the symptoms which precede death being febrile and similar to those indicative of hepatic disease,—such as heat of skin, anxiety of countenance, nausea, anorexia, indigestion, pain, or a sense of weight and fulness in the hepatic region, oppression across the epigastre, watchfulness, and disordered secretions. This black colour is sometimes confined to the surface, and sometimes pervades the substance of this organ, and an additional proof of its phlogistic nature is found in the fact, that it is occasionally combined with abscess and lymphatic deposits upon the liver. An instance of this kind is given by Portal, (*Maladies du Foie*) and the following is detailed by the author;—

A gentleman, æt. 22, was, on the 15th of June, siezed with pain of epigastre and vomiting, his pulse was frequent, and pressure aggravated his uneasiness. The disease being taken for gastritis, bleeding, blistering, and purgatives were actively employed, which abated the vomiting and pain, but on the 18th jaundice appeared, and he was visited by the Dr. on the 20th. Pulse was then 96 and soft, bowels were open, and there was little pain except on pressure over the liver; on the 21st there was no change; on the 22d the pulse rose to 140; on the 23d it was 160, there was more fever, restlessness, and some tension in the region of the left lobe. On the 24th he continued in the same state in the face of every treatment, and on the 25th he died.

*Inspection.* The left lobe of the liver, and the adjoining portion of the right contained several small abscesses full of pus, the hepatic and common ducts were obstructed by large calculi, and the entire substance of the liver was soft, broken down, and of a very dark, or nearly black colour. The other viscera were healthy.

Perhaps there is no disease which forms more slowly or more silently than *hepatic abscess.* Years may be consumed in its maturation, and the most observant experience may fail to detect it when formed. It may be the termination of a chronic action, which had succeeded more acute disease and had escaped all notice, or it may come on without being preceded by any acute symptoms, and without being at all suspected. In a case of the first kind, mentioned by Hasenoehrl, the disease ended fatally six months after the acute symptoms had disappeared, and the liver was found to be nothing more than a large cyst filled with offensive pus. In other cases, however, the disease is not so concentrated, but is divided into several small abscesses without any communication, being lined with false membrane. We have seen crude tubercles in this stage of softening, enveloped in the parenchyma of the liver, and it is probable, from the imperfect description annexed to them, that many of the cases considered to have been specimens of isolated abscess, were nothing more than softening collections of tubercular matter. When the diseased portion of the liver contracts adhesions with the surrounding parts, nature frequently contrives an outlet through which the contents of the abscess are discharged; and in this way it may be emptied into the stomach, intestines, or upon the external surface. In a case by Malpighi, the biliary duct acted as a duct for the abscess, and in another by Bonetus, the patient voided by stool large quantities of a fluid similar to what was contained in the cyst. But, perhaps, the most extraordinary passage which nature devises for its escape is through the mouth, by the formation of adhesions, *per medium diaphragmatis*, between the liver and lungs. Numerous instances of this kind are on record; the following, we presume, will be considered one of them:—

A lady, æt. 40, after being affected for some months with uneasiness in the hepatic region, was seized on the 5th of Nov. with violent pain in that situation, and vomiting. By proper treatment these symptoms became, for a time, more mild, but they soon returned, complicated with fits resembling syncope, and reduced her to a great degree of weakness. The region of the liver was tense, tender, and enlarged, the pulse small and frequent, and in the end of Dec. cough and purulent expectoration supervened. On the 14th of Jan. during a violent fit of coughing, she expectorated about ij℔ of pus, on the 15th j℔, and about the same quantity on each of the two following days. From this time until the 25th the expectoration considerably diminished, when she again brought up about j℔, and the same quantity a few days after. The tension and enlargement of the liver, which had been gradually decreasing, was now completely removed, and, with the exception of a trifling expectoration of purulent matter which only continued a few weeks, all her symptoms disappeared, and by the end of May she was free from complaint.

The Dr. describes three varieties of *ramollissement of the liver*. In the first, or simple form, the parenchyma is friable and soft, without exhibiting any change of colour, and the peritoneal coat is either separated, or easily separable from the diseased part, which is sometimes infiltrated with sanious fluid, and can be broken down by the finger without much force. This appearance is most frequently observed on the convex surface of the liver, and being accompanied with abscess and other inflammatory results seems to arise from phlogistic action. It is reported by Mr. Annesley to be very common in India, and we have seen many mild cases in the bodies of those who have sunk under typhus fever. In the second form, the texture and consistence of the liver are still more injured, and the softened substance is converted into a dark mass, which bears some resemblance to a soft coagulum of venous blood. There is every reason to believe, that this is a consequence of inflammation, and that it is analogous to gangrene in muscular tissue. In a case by Boisment, the symptoms were chiefly vomiting and an icteroid condititi on of skin, so that it does not appear to be a disease of much activity. The white, or encephaloid ramollissement of the liver, constitutes the third and last variety, but, as little is known either of its etiology or symptoms, the accompanying history will be read with interest:—

A gentleman, æt. 65, was attacked, in Sept., with diarrhœa, and pain in the hepatic region, which was much aggravated by motion. On the 1st of Oct. his fever became so severe, that bleeding and blistering were found necessary, and, although such treatment removed the more acute symptoms, he still complained of pain in his right side, had some cough and mucous expectoration. Mercury was tried with transient improvement, but in the end of Nov. he began to emaciate, and the pain of his right side returned. In Dec. anasarca of the limbs appeared, the pulse became frequent and weak, and the pain continued fixed about the right lobe of the liver; yet nothing was discoverable upon pressure, and there was no jaundice. On the 4th of Feb. he died.

*Inspection.* The liver was scarcely above the natural size, but its edge was firmly attached to the arch of the colon; externally it was closely covered with a crop of semi-transparent tubercles, the largest of which did not exceed the size of a split-pea; internally it was soft, of an almost pulpy consistence in some places, and of a white or ash-colour throughout, resembling brain. Other viscera healthy, but there was some water in the peritoneal sac.

### CHRONIC DISEASES OF THE LIVER.

*Chronic Inflammation* may supervene on an acute attack, or occur as a primary affection; in either case the principal symptoms are functional. There is usually a sense of pain or weight in the right hypochondre, severe dyspeptic symptoms and emaciation, often vomiting and sometimes jaundice, the bowels are slow, and a dragging pain is often felt in the right shoulder, fever approaches towards evening and the night is restless. If an examination be made, some enlargement will be frequently observed, but it is not a rare occurrence when nothing morbid can be discovered. After death, however, more or less hypertrophy is usually found, and more especially in the right lobe; the substance is usually dark or variegated with lines of a lighter colour, its consistence may be denser or softer than na.ural, and abscesses or tubercles occasionally occur. Simple enlargement without change of texture is a rare occurrence in adults, young persons of a scrofulous habit being most obnoxious to it; and it is a curious circumstance that it sometimes assumes an intermittent character, disappearing and returning at uncertain intervals. In a patient, mentioned by Andral, who was labouring under diseased heart, his liver was distinctly felt to become enlarged during the paroxysm, and to subside into its ordinary volume as soon as it was over. Such augmentation is produced by congestion, and must be, therefore, temporary; but when the enlargement becomes permanent, gastric disturbance is soon occasioned, and jaundice, or dropsy not unfrequently supervenes. This permanent change of volume may be effected in various ways;—it may consist of tubera upon its surface, or within its substance;—of tubercles in every variety of stage and form;—of hydatids and cysts containing a watery fluid, and covered by its peritoneal coat; or, finally, of an entire alteration of structure and aspect.

When the liver is enlarged by tubera, the symptoms are exceedingly obscure, unless the tubera be numerous, or the intervening hepatic structure be likewise unhealthy. These tubera exhibit a variety of textures, being sometimes fibrous, again tubercular, and frequently little better than cysts filled with a viscid fluid. The succeeding case will illustrate some of these:

A dyspeptic gentleman, æt. 67, began to complain of uneasiness in the chest, loss of strength and emaciation, which were much diminished by a visit to the country; but in May he became worse, a severe pain settled in his back, which rendered exercise fatiguing and his nights sleepless. In June, his emaciation was considerable although his appetite and digestion continued good, and the pain of back remained unabated. On examination, however, nothing could be discovered near the seat of pain, but in the abdomen a mass of disease was felt, extending from the ribs nearly to the spine of the left ilium, which was not painful under pressure, was never before noticed, and could not be traced to any origin. His strength now sunk rapidly, but his appetite was tolerable, and his bowels natural until a few days before death, when nausea, thirst, and other unfavourable symptoms announced the approach of dissolution.

*Inspection.* The entire liver, but its left lobe especially, was much enlarged, externally it was dark and variegated, internally composed of round tubera of the size of oranges, of a whitish colour, some approaching to the hardness of scirrhus, others were softer, and some contained a puriform fluid.

*Hydatids* are of frequent occurrence in this organ, but in their effects upon the general system produce no symptoms by which their presence can be distinguished. They may either be imbedded in its substance, or attached to its surface in cysts, which are sometimes lined with false membrane, and not unfrequently contain portions of bone. These cysts are often very large; Mr. Annesley describes one which contained a quart of watery fluid in which an hydatid floated; and the author details the history of another, whose cavity contained no less than xviij℔ of a transparent colourless liquid. The whole case is peculiarly interesting, but our limits prevent us from adducing it.

Under the head of *pale degeneration without much change of texture*, the author includes a class of affections which often occur, but with considerable varieties:—

"The liver so affected has lost, in a greater or less degree, the healthy appearance, and has become of a paler colour, without any considerable alteration from the healthy texture. This change, in some cases, consists merely of a much paler shade of the natural colour; in others, it is a dull white or ash colour, and frequently a uniform dull yellow, closely resembling the colour of impure bee's wax. The liver thus affected may be of the natural size, or it may be increased in size, or it may be diminished. The symptoms accompanying these changes have not been well investigated; they are chiefly observed when the patient has died of some other affection, and are scarcely themselves to be considered as fatal diseases, though there may have been symptoms indicating some derangement of the functions of the liver or the stomach. The most remarkable of these changes is the yellow degeneration of the liver, which, from its resemblance to wax, has received from the French writers the name of *Cirrhose.* It is sometimes found in irregular portions, mixed with the healthy structure, and sometimes in small nodules like peas dispersed through the substance of the liver; but, in many cases, the whole liver is found changed into one uniform mass of this appearance, exactly resembling a mass of impure wax, and it seems to possess very little vascularity. A case is described by Clossy,* in which the structure of the liver was wholly constituted of a congeries of little firm globules, 'like the the vitellarium of a laying hen;' it occurred in a boy of 15, who had immense ascites. In a case by Boisment, these nodules were as large as peas, and the liver was diminished in size; the case was chronic with ascites. The French writers have a controversy whether the Cirrhose or yellow degeneration of the liver be a new formation, or a hypertrophia of a yellow substance, which they suppose to constitute a part of the structure of the liver in its healthy state. No good can arise from such discussions, as it is impossible to decide them." 345.

When change of colour is complicated with that of structure, the disease assumes many characters, and the symptoms of each may be modified by these individual peculiarities. Thus, change of colour and structure may be accom-

* "Clossy.—Observations on some of the Diseases of the Parts of the Human Body.

panied with change of volume;—the change of structure may consist of simple condensation, confirmed induration, or cartilaginous conversion; and for the natural colour may be substituted blackness, or pallidity. Induration is frequently attended by diminished volume, as ramollissement is by hypertrophy; but this connexion is not indissoluble, as cases are recorded whose results are opposed to it. In the succeeding history we find the colour pale, the size natural, and the structure cartilaginous; but the symptoms of the first stage indicate an action too acute for a purely chronic disease:—

A man, æt. 45, was seized in May with severe pain in the epigastrium, which shortly moved into the hepatic region. It was increased by inspiration, there was some cough, and the pulse was 120. During 60 hours he was bled to 145 ozs. and his symptoms so improved that he soon went into the country; but cough, dyspnœa, and debility were still complained of, dropsical symptoms soon appeared, pain of right side increased, and he died in the beginning of August. There was much water in the abdomen, the liver was of natural size, but entirely changed in colour and texture, being of a dull white, very hard, and in some parts nearly cartilaginous. Lungs and other viscera healthy.

In a case by Andral, where debility, anorexia, pain of loins and shoulders, icteroid state of skin, and dyspnœa formed the leading features, the liver was found very small, of a scirrhous hardness, and covered with a kind of sandy matter; in another by Boulland, in which pain in the right hypochondre and jaundice were present, the liver was small, hardened, and internally presented a surface variegated with grey and yellow. But our author has given a more interesting example of this disease than has been yet recorded.

A man, æt. 40, complained of pain of right side, unaffected by breathing or pressure, and a severe cough, at first dry, but afterwards attended with copious sputa, which was often tinged with blood. Hectic symptoms not long after supervened, emaciation and general dropsy followed, and after an illness of 18 months he died exhausted. There was some water in the chest, but his lungs were sound; liver scarcely exceeded in size the *bulk of the hand half-folded*, it was drawn up under the ribs, adherent to the diaphragm, studded on its surface with tubercles, and internally pale and indurated.

We have now arrived at the Dr.'s observations upon treatment, and being very concisely given, we will transfer them to our pages in his own language.

"The real diseases of the liver resolve themselves into two great classes, the acute and the chronic. The acute affections are to be combated by the means adapted to other inflammatory diseases, namely, general and topical blood-letting, blistering and saline purgatives. In the less active cases, indicated by local pain and tenderness, without constitutional disturbance, we rely chiefly upon repeated topical bleeding, blistering, issues, free and continued purging, and a careful regulation of diet. In both cases, when the activity of the disease is subdued by these means, benefit is obtained from the cautious use of mercury; and it seems in general to be most advantageously applied by friction.

"In regard to the chronic affections of the liver, under the various forms which have been detailed in the preceding observations, it will probably be admitted that a large proportion of them are beyond the reach of any human means. The treatment of these ought to be entirely palliative, consisting of a careful regulation of the diet and the bowels, with mild tonics, &c. This I conceive to be a point of much practical importance, because these affections often exist for a long time without materially injuring the health of the patient; and by treatment entirely palliative, his life may be perhaps prolonged, and certainly rendered more comfortable. But when such cases are treated actively by courses of mercury, the strength uniformly sinks in a very rapid manner, and the patient's life is often evidently shortened. In several cases of chronic affections of the liver, accompanied by jaundice, I have seen very good effects from the external use of Iodine, in an ointment containing ʒss. to ℥j. of axunge.

"In the preceding observations I shall probably be charged with attaching too little importance to mercury in the treatment of this class of diseases, and I am well aware of the delicate ground on which I tread, when I venture to express a doubt of its adaptation to all stages and all forms of diseases of the liver. In doing so I would be distinctly understood to express myself in regard only to the liver diseases of this country, having no experience of any other; but in respect to these I have no hesitation in saying, that mercury is often used in an indiscriminate manner, and with very undefined notions as to a certain specific influence which it is believed to exert over all the morbid conditions of this organ. If the liver is supposed to be in a state of torpor, mercury is given to exite it; and if it is in a state of acute inflammation, mercury is given to moderate the circulation, and reduce its action. Effects the most indefinite, if not contradictory, are also sometimes ascribed to it in regard to its influence on the secretion of bile, and in those affections which are commonly called bilious. Upon the principles of induction with regard to cause and effect, which are recognized in other sciences, it may be doubted whether all these maxims can be right, but I will not take upon me to decide which of them is wrong. I leave the subject, therefore, with merely throwing out these doubts, the force of which must be felt by every pathological inquirer; and with hazarding the opinion, that much of the prevailing doctrine on derangements of the liver requires to be revised, and perhaps corrected. There are certainly many parts of it, of which the pathologist must be allowed to doubt, whether they are not at variance with the principles of philosophical inquiry." 361.

In an appendix, after adducing a case from Andral illustrative of *hæmorrhage from the liver*, and another from his own practice, in which the patient died of *rupture of this organ* occasioned by a fall, the author makes a few remarks upon *diseases of the bile and gall-bladder*, and upon the *pathology of jaundice*. The bile, he observes, may be either defective, or redundant in quantity; or diseased in quality. Sometimes its appearance and properties differ little from those of water, at others it is as gross as pitch; sometimes dyspepsia arises from its imperfect secretion, at others its redundancy has been taxed as the cause of bilious diarrhœa. Neither of these points, however, has been made out with certainty, and the facts in support of each are scanty and incomplete.

Impaction of the gall-bladder, or its ducts with biliary calculi, is a frequent occurrence, and rupture or ulceration of their coats is occasionally found. The latter accident is always fatal, the extravasated bile inducing the most inveterate inflammation, and although the former is usually succeeded by jaundice, a case is detailed by the Dr. which terminated fatally without being attended by any icteroid symptoms.

It has been disputed, whether biliary calculi ever form in the substance of the liver, and whether, when found in the hepatic duct, they can ever occasion icterus? But the testimony of Morgagni has settled these questions in the affirmative. While they lodge in the gall-bladder they are generally productive of little inconvenience, and such a fact is remarkable when we consider that their number has been sometimes so great as to fill up the entire of its cavity. Morgagni refers to cases in which several hundreds were found, and to one in which there were 3646. Regular exercise and constant attention to the bowels are, perhaps, the only means we have of preventing their formation, and when their presence gives rise to urgent symptomstheir passage must be promoted by opiates, warm bath, laxatives, and perhaps injections of tobacco. The author should have added, that local, and even general bleeding will be sometimes necessary.

Jaundice is often dependent on hepatic inflammation, existing in an obscure form and to a small extent. Such a cause may be suspected when there is much tenderness in the region of the liver, without fever or general symptoms. If the inflammation be considerable, jaundice is not a common consequence, except when the concave surface is affected.

"It appears, however, that there is a state of the liver which gives rise to jaundice, and which does not amount to inflammation, though it is evidently allied to it. The circumstances, under which we are chiefly able to trace this affection, are when jaundice appears in connection with inflammation of the lower part of the right lung. In a case of this kind, which had been accompanied by the usual symptoms of pneumonia, with the addition of violent hiccup, I found an abscess of the lower part of the lung in contact with the diaphragm, but could not detect any appearance of disease in the liver, except that it seemed to be rather paler than usual on the surface. Bonetus relates a similar case in which the disease was in the lungs, the liver being merely paler than natural. There had been fever with convulsions, and death in 15 days. It is probable, therefore, that the liver may be affected, in a manner analogous to that now referred to, from other causes which in a great measure elude our observation. To this principle we may perhaps refer some of those temporary cases of jaundice which appear to arise from disorders of the bowels,—also those cases which seem to be induced simply by external heat, and have been ascribed to overflow of bile. Jaundice is also occasionally observed in connection with disease of the heart, arising probably from the impeded return of the blood from the liver; and it has been known to supervene upon suppression of the hæmorrhoidal discharge and other evacuations which had become habitual. Portal has seen it supervene upon suppression of leucorrhœa; and he also mentions a woman who had been long affected with a copious and very fetid discharge from the arm-pits, and immediately became jaundiced, when she suppressed it by means of a preparation of alum." 372.

When jaundice arises from inflammation, the general remedies against such action must be adopted, and after the active symptoms have been overcome further relief may be obtained from friction with mercury, or iodine.

Dr. Marsh (*Dublin Hospital Reports, vol. III.*) has endeavoured to prove, that mucous inflammation of the duodenum has produced jaundice by closing up the mouth of the biliary duct, and others have asserted that collections of hardened fæces within the colon have given rise to the same effect. However this may be, there is no doubt but icterus occasionally depends upon intestinal derangement, and that enlargement of the pancreas, and diseases of the pylorus may occasion the absorption of bile.

It is questioned by the Dr. whether injuries of the head ought to rank among the causes of this disease; that they have been contemporaneous is certain, but this circumstance affords too feeble a presumption of their etiological relation, seeing that the same accident which injured the head may have disordered the biliary apparatus; but mental disquiet will certainly produce it; such cases are recorded by almost every writer upon this subject, and they furnish a striking *reverse* illustration of the sympathies which hold between the liver and brain.

### PATHOLOGY OF THE SPLEEN.

The diseases of the spleen are very similar to those of the liver, but they are less frequent in their occurrence and less important in their influence. The rank which this organ holds in the economy of life has not as yet been ascertained, the functions it is destined to perform are not well understood, and the diseases to which it is obnoxious have neither been carefully studied, nor clearly revealed. Our ignorance of its use during health has rendered us less alive to its conditions in disease, and the obscurity of its functions when natural, has made their study, when disordered, less interesting in its object, and less successful in its issue. But, like every other organ in the body, it is subject to inflammation, and inflammation may terminate in the ordinary results of such action upon such texture;—in suppuration, ramollissement, hypertrophy, and induration. Besides these, it is not unfrequently affected with tubercles and hydatids, and it may give rise to hæmorrhage, or be lacerated, as the liver, by external violence.

We believe that *active inflammation* of the substance of the spleen has been seldom noticed, but whether this depends upon its singularity or obscurity has not been exactly ascertained. Portal describes an instance in a man who died of fever, in which pain of the left side, cough, dyspnœa, and palpitation were the accompanying symptoms. Our author has not witnessed any idiopathic case, but suspects that the symptoms are in general most obscure, when the inflammation is seated in the peritoneal coat. *Chronic inflammation* is, however, less rare, but its phenomena are obscure and its duration protracted.

*Suppuration* seems also to be a rare occurrence, and the cases which have been recorded of it are few and unsatisfactory. M. Jacquinelle (*Journal de Med.*) relates one in which the young man complained of pain and fulness in the left hypochondre, palpitation, faintings, and emaciation. Before death the pain subsided, and a quantity of dark fetid matter was discharged by stool. The spleen was found much enlarged, and contained an abscess that had burst into the colon. Grotanelli records another, in which the abscess burst into the peritoneal cavity, and proved fatal in three days; M. Cazé another, which opened into the stomach, and was marked by vomiting of bloody pus and a sense of palpitation in the epigastre; and Heide another, which communicated with the external surface by a fistulous ulcer which extended from the spleen to the umbilicus, betwixt the peritoneum and abdominal muscles. These abscesses occasionally obtain a remarkable size; in the *Mem. of the Acad. of Sciences* instances are given, where viij. lb. were drawn off by tapping, and where xxx. lb. were found after death. In some of the Walcheren cases of fever Mr. Wardrop found the spleen converted into a mere cyst of puriform fluid.

*Ramollissement* is a very common appearance after some forms of general disease. In fatal cases of genuine typhus we believe it will seldom be found absent; and the Doctor considers it the only morbid result, when the patient dies with obscure and protracted symptoms of diseased spleen. In this affection the substance of the spleen is broken down into a semi-fluid resembling melted pitch, or black-currant jelly, which is rather contained within its peritoneum as a bag, than covered by it as an investment. It is sometimes attended by inflammatory symptoms and connected with inflammation of other organs, but more frequently it is unaccompanied by much constitutional sympathy, and proceeds unmarked by any very palpable phenomena. A man mentioned by Sennertus, after complaining for some weeks of loss of appetite and pain in the left side, was seized with bloody diarrhœa, and died in a few days. Upon inspection the principal disease was in the spleen, which had degenerated into a bag of dark fetid matter like the lees of oil.

When the spleen is *enlarged* its vascularity is much increased, but its structure may remain unchanged, and the general health almost undisturbed. A woman is mentioned by Lieutaud, who had lived for 17 years with an enlarged spleen, that weighed after her death 32 pounds; and Dr. Crane observes, that the inhabitants of Lincolnshire are often affected with it for 20 years, without exhibiting much appearance of disease beyond a pale or yellowish aspect. In some cases, nevertheless, its effects are troublesome, disordering the bowels, deranging the stomach, destroying the strength, and favouring local hæmorrhage, or general dropsy.

"One of the most singular facts in the pathology of the spleen, is the very rapid manner in which enlargement of it takes place, and the equally rapid manner in which it subsides. Some of the cases of this kind which I have seen, appeared so very extraordinary, that I suspected some fallacy, until I found similar cases described as of frequent occurrence by writers on the diseases of India. Several years ago, I saw, along with Dr. Combe of Leith, a seaman who had contracted ague in England a few weeks before, and had returned to Leith with the disease going on in the usual manner. In the left hypochondrium, there was a firm defined tumor arising from beneath the margin of the ribs, and projecting downwards several inches. We agreed that our first object was to arrest the fever by the usual means, leaving this remarkable tumor for future consideration; but, on returning about a week after, I found that the fever had been easily arrested, and that the tumor was entirely gone." 385.

From the circumstance of remittent and intermittent fever being the most frequent cause of this disease, mercury was once largely employed in its treatment; but the effects which it often produced, in mortifying the mouth and sinking the strength, have lately discouraged its exhibition, and at present it is seldom thought of. If there be much tenderness in the region of the spleen, or symptoms of general excitement, topical bleeding should be followed by blistering, or a seton, and purgatives should be freely administered.

The natives of India apply the actual cautery and use a combination of aloes, garlic and vinegar; and the spleen powder of Bengal, which is described as endowed with almost specific virtue, is composed of rhubarb, jalap, scammony, and cream of tartar, with calomba powder and sulphate of iron.

*Tubercles and Hydatids* are frequently found in this organ; the former generally in connexion with tubercular disease in other parts, and the latter in cysts formed by its peritoneal coat. But neither is characterized by any peculiar symptoms, and cannot, therefore, be the subject of any specific treatment.

*Pale induration* of the spleen is mentioned by Portal and Lieutaud, and *black induration* by Diemerbroeck; but the author has not met with either. Fournier relates a case of *fatal hæmorrhage* from this viscus, and Lieutaud, Tulpius, and Chisholm furnish examples of rupture by external violence. In addition to these diseases, the Doctor mentions infiltration of the substance of the spleen with a gelatinous fluid, deposition of fatty matter into its structure, an ossified or cartilaginous state of its external surface, diminution of size, and a stony degeration of its texture; but to enter into any detail of such affections would be fruitless.

### PATHOLOGY OF THE PANCREAS.

Inflammation, ending in suppuration and gangrene, enlargement, induration, and calculous deposition, are the principal morbid conditions to which the pancreas seems liable; but our knowledge of either is very imperfect, and the symptoms of each ill-defined and obscure. Tulpius, Bartholinus, Guido Patin, Portal, Baillie, and Percival, have published cases of suppuration of this body, but the attending symptoms were exceedingly different. In one there were general pain of abdomen, spasm of abdominal muscles, nausea, a tendency to diarrhœa, and dropsy;—a second had jaundice and bilious vomiting, a tumour was perceptible in the epigastre, and bloody pus was discharged by stool;—and, in a third, death was preceded by three or four attacks of vomiting and syncope. A *sphacelated* state of this organ was found by Barbette as the only morbid appearance in a man who died of urgent vomiting after a short illness, and the same appearance occurred in a man mentioned by Greizil, who had been addicted to colic and died rather suddenly, complaining merely of a sensation of internal cold. The following case will illustrate *scirrhous induration* of the pancreas; and the next *enlargement* with a mixed state of disease.

A woman, æt. 40, complained of epigastric uneasiness and frequent vomiting, which would not permit any food to remain upon her stomach. In about a year she died without any other symptom, and, although quite exhausted, the integuments of her body were covered with fat, and those of the abdomen were coated with a layer two inches thick! The pancreas was found in a state of uniform scirrhus, without much enlargement. No other disease could be discovered.

A man, æt. 26, complained of pain in the left hypochondre, oppression at the stomach, indigestion, and gradual emaciation. But he had no vomiting, his bowels were very tractable, and external examination could detect no disease. After two years' continuance of these symptoms he died without any alteration in his case, except that jaundice had appeared a few days before death. The seat of the pancreas was found occupied by a mass, four or five inches broad and somewhat less in thickness, which adhered to the spine, and surrounded the aorta. It was partly composed of cartilage, and partly of alternate layers of yellowish and white matter, resembling medullary sarcoma. The other organs were healthy, except the liver, which was rather pale and somewhat enlarged.

*Calculi* have been found in the pancreas by De Graaf, Portal, and others. They seem to consist of carbonate of lime, and they vary in size from that of a pea to a hazel nut. In a man, who died of diseased aorta, Portal found twelve imbedded in this organ, which was likewise much enlarged; so that, like gall-stones in the gall-bladder, they may exist in great numbers without deranging the patient's health to any perceptible degree. Indeed, the same remark is applicable to most of the chronic diseases of this viscus.

"Many cases are on record of chronic disease of the pancreas, exhibiting the same diversity of symptoms which occurred in the examples now described, and nearly in the following proportion. Of twenty-seven cases which I find mentioned by various writers, six were fatal with gradual wasting and obscure dyspeptic complaints, without any urgent symptom. In eight, there was frequent vomiting, with more or less pain in the epigastric region; and thirteen were fatal, with long continued pain without vomiting. In some of these, the pain extended to the back; and in others, it was much increased by taking food. In several, there were dropsical symptoms; and in three or four there was jaundice from the tumor compressing the biliary ducts. In the morbid appearances, also, there was great variety; the pancreas being in some of the cases much enlarged, in others, in a state of scirrhous hardness with very little enlargement. It does not appear that any distinct relation can be traced betwixt the urgency of the symptoms and the degree of enlargement; for this existed in a great degree in some of the cases in which the symptoms were slight and obscure; and there was hardness with little or no enlargement in others, in which the symptoms were defined and violent." 396.

We have now faithfully followed our author through his Pathology of the Stomach, Duodenum, Liver, Spleen, and Pancreas, and have furnished our readers with ample materials for reflection upon a series of most interesting diseases until the appearance of our next, when we hope to resume the subject, and give an attentive digest of his Intestinal Pathology. Till that period we decline making any general criticisms, convinced that the diligence which we have displayed in the execution of our present article, and the spirit in which it has been written, will intelligibly communicate our sentiments of the author without the aid of direct eulogy, and will ensure for his re-appearance a cordial welcome.

